# The structure of WbnH in a near active state

**DOI:** 10.1007/s13238-015-0151-7

**Published:** 2015-04-01

**Authors:** Fengzhi Li, Siwei Li, Xiaofen Liu, Xue Yang, Peng Wang, Yuequan Shen

**Affiliations:** 1 State Key Laboratory of Medicinal Chemical Biology, Nankai University, Tianjin, 300071 China; 2 College of Life Sciences, Nankai University, Tianjin, 300071 China; 3 Tianjin Institute of Industrial Biotechnology, Chinese Academy of Sciences, Tianjin, 300308 China; 4 Synergetic Innovation Center of Chemical Science and Engineering, Tianjin, 300071 China

**Dear Editor,**

Gram-negative bacteria possess a complicated membrane system that plays an essential role in interactions between bacteria and the environment (Reeves and Wang, 2002). The inner leaflet of the membrane is composed of various glycerophospholipids, and the outer leaflet consists primarily of lipopolysaccharide (LPS) molecules.

LPS serves as a barrier to protect bacteria against adverse environmental elements and is thus necessary for bacterial growth (Raetz and Whitfield, 2002). LPS consists of a lipid A moiety, by which LPS is anchored to the membrane, a core oligosaccharide, which extends outward from the membrane, and a distal O-specific polysaccharide chain (O antigen) (Raetz and Whitfield, 2002). The core oligosaccharide is typically strain-specific, while the O antigen is serotype-specific. In addition, bacteria often mimic human polysaccharide processing by generating O-antigenic polysaccharides that are structurally similar to human polysaccharides in order to evade the human immune system. This mimicry can lead to infections and serious consequences for humans (Jones, 2005). Thus, the development of vaccines that inhibit the generation of bacterial O antigen is urgently needed (Yi et al., 2003).

O-Antigen biosynthesis has been studied for several decades. O-Antigenic polysaccharides consist of one to ten sugars with up to 50 repeating oligosaccharide units. Two major O-antigen biosynthetic mechanisms have been described; these mechanisms diverge primarily in O-antigen translocation and polymerization (Hug and Feldman, 2011). One of these mechanisms is the Wzy-dependent pathway. First, O-units are sequentially assembled onto lipid carriers in the cytoplasm. Second, these O-units are translocated across the inner membrane to the periplasm via the flippase Wzx. Finally, the O-antigen polymerase Wzy catalyzes the polymerization of O-units from the sugar-reducing end and the enzyme Wzz simultaneously controls the chain length of the polysaccharide (Woodward et al., 2010). The majority of heteropolysaccharides are synthesized via this mechanism. The second mechanism is the ABC transporter-dependent pathway. In this pathway, the O-units are completely assembled in the cytoplasm, and the polymers are then translocated across the membrane by the ABC transporter (Linton and Higgins, 1998).

In the Wzy-dependent pathway, the first reaction transfers a sugar phosphate from the nucleotide sugar donor substrate to the carrier lipid undecaprenyl phosphate (Und-P). This reaction is typically processed by the sugar phosphate transferase WecA and its orthologs (Touze et al., 2008). Specific glycosyltransferases catalyze the sequential addition of different sugars onto the Und-PP-sugar after initiation (Hug et al., 2010). WbnH is one of these glycosyltransferases. WbnH catalyzes the formation of the GalNAc α-1,3-GalNAc structure that precedes the tetrasaccharide fragment in the O-unit of *E. coli* O86 (Yi et al., 2006). In addition to WbnH, WbnJ, WbnK and WbnI also contribute to the sequential generation of the repeating unit pentasaccharide-PP-Und (Woodward et al., 2010). Glycosyltransferases have distinct specificities that correspond to different donors and substrates. The pyrophosphate and lipid portions of the substrates are essential elements for WbnH activity. The WbnH acceptor naturally contains a 55-carbon undecaprenyl hydrophobic chain. However, the chemically synthesized 11-carbon lipid chain GalNAc α-PP-O(CH2)11-OPh can also act as a substrate of WbnH *in vitro* (Yi et al., 2006).

The majority of glycosyltransferases are classified into the GT-A and GT-B superfamilies based on their fold types and catalytic mechanisms (Charnock and Davies, 1999). WbnH belongs to the GT-B superfamily, as it does not require a metal ion for activity (Albesa-Jove et al., 2014). Thus, WbnH does not contain the canonical DXD motif for binding divalent metal ions (Albesa-Jove et al., 2014). However, the exact catalytic mechanism of the WbnH glycosyltransferase is currently unclear. Here we report the crystal structure of WbnH and provide a structural foundation for explorations of the molecular mechanism by which WbnH adopts acceptors and substrates.

The crystal structure of WbnH from *E. coli* O86:H2 was determined at a resolution of 2.2 Å using the single-wavelength anomalous dispersion (SAD) methodology. The overall structure of WbnH displays the typical fold of GT-B glycosyltransferases. The structure consists of two Rossmann fold-like lobes: the N-lobe and the C-lobe (Fig. 1A). Both of these lobes are composed of a core of seven or eight parallel β-strands surrounded by α-helices. The N-lobe includes residues 1–166 (β-strand 1–8 and α-helices 1–8) and residues 323–338 (α-helix 15), and the C-lobe includes residues 167–322 (β-strand 9–13 and α-helices 9–14). The N-lobe and C-lobe are separated by a deep cavity, which presumably accommodates the donor and the acceptor for the enzymatic reactions.

**Figure 1:**
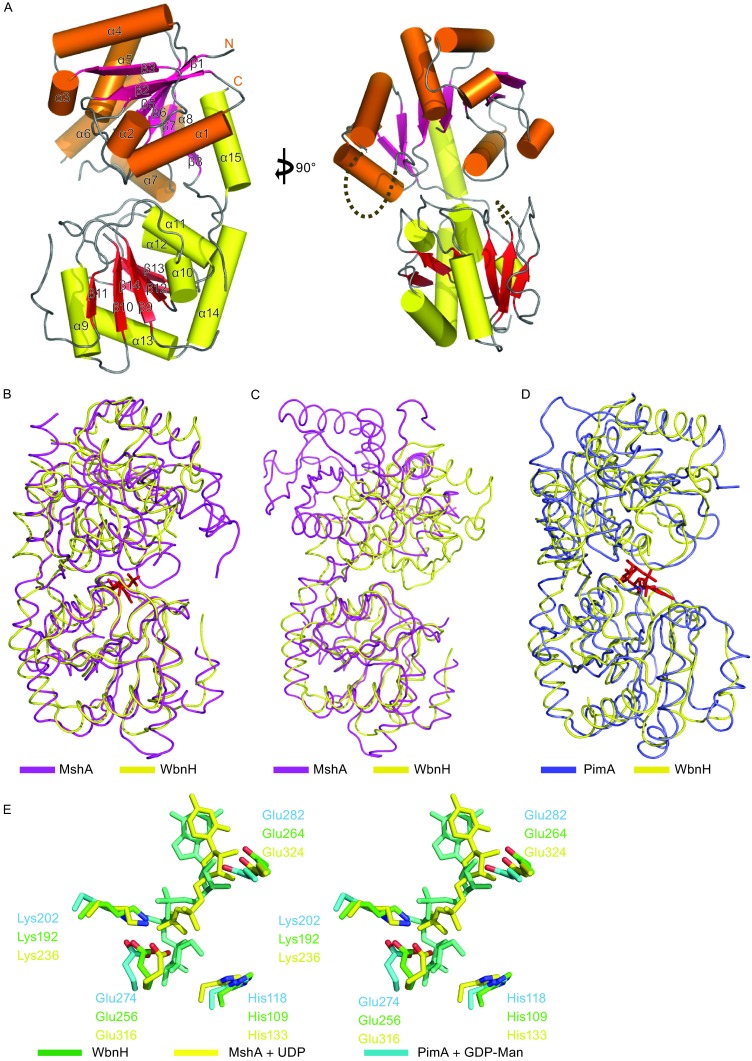
**WbnH in a near active state**. (A) Cartoon representation of the overall structure of WbnH. The α-helix and β-sheet in the N-lobe are presented in orange (except α-helix 15, which is presented in yellow) and pink, respectively. The α-helix and β-sheet in the C-lobe are presented in yellow and red, respectively. (B) Structural comparison of WbnH and MshA in complex with UDP (PDB code 3C4Q). WbnH is presented in yellow, and MshA is presented in purple. The UDP substrate of MshA is represented as red sticks. (C) Structural comparison of WbnH and MshA without substrate (PDB code 3C48). WbnH is presented in yellow, and MshA is presented in purple. (D) Structural comparison of WbnH and PimA in complex with the donor substrate GDP-Man (PDB code 2GEJ). WbnH is presented in yellow, and PimA is presented in blue. The GDP-Man substrate of PimA is represented as red sticks sticks. (E) Stereo view of an overlay of the conserved donor substrate binding sites of WbnH, MshA with UDP and PimA with GDP-Man

To further explore the details of the working mechanism of WbnH, we performed structural homology searches using the DALI server. The results revealed that the WbnH structures are highly similar to several previously reported GT-B enzymes, although the protein sequence homology is quite low (Fig. S1). The most similar of these enzymes is the *Corynebacterium glutamicum* glycosyltransferase MshA-UDP·1-_L_-Ins-1-P complex (DALI Z-score of 32, r.m.s.d. of 2.7 Å and 19% sequence identity), followed by a GT-B family glycosyltransferase from *Bacillus anthracis* ORF BA1558 that is termed BaGT4_BA1558_ (DALI Z-score of 31.3, r.m.s.d. of 3.2 Å and 18% sequence identity), the PimB’ phosphatidylinositol mannosyltransferase from *Corynebacterium* *glutamicum* in complex with GDP-man (DALI Z-score of 28.3, r.m.s.d. of 3.6 Å and 18% sequence identity) and the phosphatidylinositol mannosyltransferase PimA from *Mycobacteria* in complex with GDP-man (DALI Z-score of 28.3, r.m.s.d. of 3.1 Å and 16% sequence identity). A multiple sequence alignment among these proteins revealed that WbnH shares a low sequence identity of no more than 20% with these enzymes; however, residues around the active site are quite conserved (Fig. S1).

The superposition of the structure of MshA in complex with UDP (PDB code 3C4Q) onto WbnH revealed a similar overall structure (r.m.s. deviation of 2.7 Å for 314 of 393 Cα pairs) (Fig. 1B). In contrast, a structural comparison of MshA without substrate (PDB code 3C48) to WbnH revealed a large difference, particularly in the C-lobe (Fig. 1C). In addition, residues 16–22 of the MshA structure without substrate are disordered but become well-ordered upon binding of the UDP substrate. In contrast, the corresponding region of WbnH (i.e., residues 7–15) formed a loop that was located in the cavity even in the absence of substrate. These results indicate that the WbnH structure is close to the active conformational state of substrate binding glycosyltransferases (i.e., in a “near-active” conformation). To further confirm this hypothesis, we performed a structural superposition of PimA alone (PDB code 4N9 W) and in complex with the donor substrate GDP-Man (PDB code 2GEJ) onto WbnH. The results demonstrate that WbnH structure is more close to the substrate binding PimA than apo-PimA (DALI Z-score of 28.3 versus 26.4) (Figs. 1D and S2).

We then investigated the residues that are critical for substrate binding in the catalytic cavity (Fig. 1E). Four residues (i.e., His133, Lys236, Glu316 and Glu324) in the MshA structure were previously demonstrated to be responsible for substrate binding (Vetting et al., 2008). The corresponding residues (i.e., His118, Lys202, Glu274 and Glu282) in the PimA structure are conserved and in very similar positions (Guerin et al., 2007; Ruane et al., 2008). In the WbnH structure, four conserved residues (i.e., His109, Lys192, Glu256 and Glu264) are located in similar positions to those of MshA and PimA, further suggesting that the WbnH structure is in a “near-active” conformation.

To obtain the details of substrate binding, we performed a co-crystallization of WbnH with UDP-GalNAC. However, this co-crystallization failed. Because the acceptor is not commercially available, we used a docking technique to determine the binding mode of WbnH with its acceptor and donor. A comparison with substrate-bound MshA revealed that the donor UDP-GalNAC was manually docked into WbnH (Fig. S3A). Four conserved residues (i.e., His109, Lys192, Glu256 and Glu264) surround the proposed position of UDP-GalNAC (Fig. S3B). Through a comparison with the structure of MshA in complex with both UDP-GalNAC and the acceptor analog 1-_L_-Ins-1-P, we are able to pinpoint the WbnH structure in complex with both the donor and the acceptor (Fig. S3C and S3D). The donor UDP-GalNAC binds primarily to the C-lobe, and the acceptor binds to the N-lobe. Moreover, one end of the acceptor is buried in the active site cavity while the other end of the acceptor is exposed in the flexible solvent region.

In this paper, we report the crystal structure of WbnH in the absence of substrate. The overall topology of WbnH is quite similar to previously reported structures of other proteins in the GT-B glycosyltransferase family. Unexpectedly, the conformation of WbnH without substrate is close to the structures of other glycosyltransferases with their substrates, especially the structure of CgMshA in complex with the UDP·1-_L_-Ins-1-P complex. Moreover, the key residues for substrate binding (i.e., His109, Lys192, Glu256 and Glu264) are highly conserved in both the protein sequence and the three-dimensional structure. These results strongly suggest that our WbnH structure is in a “near-active” conformational state.

WbnH has been proposed to transfer GalNAc from UDP-GalNAc to the C3 hydroxyl of GalNAc-PP-C55 in bacteria (Yi et al., 2006). Due to the lack of commercial availability and the difficult synthetic process of the natural acceptor for the WbnH enzyme, we were unable to explore the catalytic mechanism biochemically. Instead, we manually docked both the donor substrate UDP-GalNAC and the acceptor substrate analog 1-_L_-Ins-1-P into the WbnH structure based on structural conservation. UDP-GalNAC primarily binds to the C-lobe and is surrounded by four conserved residues (i.e., His109, Lys192, Glu256 and Glu264). The acceptor analog 1-_L_-Ins-1-P binds to the N-lobe, and the tail portion extends into the solvent region in the model; this finding is consistent with the properties of the natural acceptor GalNAc-PP-C55, which has a long tail. The model we provided here may contain some inaccuracies due to technical limitations. Nevertheless, this model provides useful clues for exploring the mechanism by which WbnH catalyzes the transfer of GalNAc from UDP-GalNAc to the GalNAc-pyrophosphate-lipid acceptor.

## Electronic supplementary material

Below is the link to the electronic supplementary material.

(PDF 372 kb)
