# Supplementary material for: The structure of WbnH in a near active state
Source: Protein Cell. 2015 Apr 1;6(8):615–8. doi: 10.1007/s13238-015-0151-7 (PMC4506291; doi:10.1007/s13238-015-0151-7)
Supplement: Supplementary file 1 — (PDF 372 kb) [file 13238_2015_151_MOESM1_ESM.pdf]

Supplemental Methods

Protein expression and purification

The *WbnH* gene was PCR amplified from *Escherichia coli* O86:H2 and then cloned into the PET-15b vector. The correct constructs were subsequently transformed into *E. coli* BL21 (DE3) and overexpressed via induction with 0.15 mM isopropyl-1-thiogalactopyranoside (IPTG) at 18 °C for 12 h.

The harvested cells were resuspended in buffer A (50 mM Tris-HCl, pH 8.0, 200 mM NaCl) and lysed via sonication. After centrifugation at 18,000 ×*g* for 40 min, the supernatant was loaded onto Ni-NTA affinity columns and washed with buffer A. The column was subjected to individual washes with 5 column buffer B (50 mM Tris-HCl, pH 8.0, 200 mM NaCl and 10 mM imidazole) and 5 column buffer C (20 mM Tris-HCl, pH 8.0, 200 mM NaCl and 25 mM imidazole). The target protein was eluted using buffer D (50 mM Tris-HCl, pH 8.5, 200 mM NaCl and 250 mM imidazole). The eluted protein solution was loaded onto a desalt column (GE Healthcare) and eluted using buffer E (20 mM Tris-HCl, pH 8.5, 50 mM NaCl, 1 mM EDTA and 1 mM DTT). The solution was then loaded onto a Q anion-exchange column (GE Healthcare) and eluted using a NaCl gradient (100-1,000 mM). Fractions of interest were pooled and loaded onto a superdex200 gel-filtration column (GE Healthcare) with buffer F (20 mM Tris-HCl, pH 8.5, 200 mM NaCl, 1 mM EDTA and 1 mM DTT), and peak fractions were identified via SDS-PAGE and pooled.

The Se-Met derivative protein was produced using the same protocol that was used for the wild-type protein, except cells of the methionine auxotroph *E. coli* B834

(DE3) and minimal medium were used to express the recombinant protein.

Crystallization and data collection

The WbnH protein was crystallized by combining 1 μl of protein solution (2.0 mg/ml) (20 mM Tris-HCl, pH 8.5, 200 mM NaCl, 1 mM EDTA and 1 mM DTT) with an equal volume of well solution containing 1 M Li_2SO_4, 0.5 M $(NH4)_2SO_4$, 0.1 M sodium citrate tribasic dehydrate, pH5.6. Crystals were obtained at 4 °C via sitting-drop vapor-diffusion.

The wild-type data were collected on the Shanghai Synchrotron Radiation Facility BL17U1 beam line, and all data were processed using the HKL2000 software (Otwinowski, 1997). Single wavelength anomalous data were collected for Se-Met substituted crystals at element SE peak wavelength on the beam line BL-17A at the Photon Factory (Tsukuba, Japan).

Structure determination and refinement

The structure of WbnH was determined using the single-wavelength anomalous dispersion methodology. The initial phases were then calculated using PHENIX Autosol (Adams et al., 2010). The models were built using the COOT program (Emsley and Cowtan, 2004). After the initial model was built, iterative refinement was performed using the PHENIX refinement program and COOT. The orientations of the amino acid side-chains and bound water molecules were modeled based on $2\boldsymbol{F}_{obs} - \boldsymbol{F}_{calc}$ and $\boldsymbol{F}_{obs} - \boldsymbol{F}_{calc}$ difference Fourier maps. The final structure had an $\boldsymbol{R}_{work}$ value of 20.48% and an $\boldsymbol{R}_{free}$ value of 23.96%. The detailed data collection and refinement statistics are summarized in Supplemental Table SI. All of the figures were

generated using the program PyMol (DeLano Scientific, LLC.).

Accession numbers

Atomic coordinates and structure factors for the reported crystal structure have been deposited in the Protein Data Bank with accession code 4XYW.

References

Adams, P.D., Afonine, P.V., Bunkoczi, G., Chen, V.B., Davis, I.W., Echols, N., Headd, J.J., Hung, L.W., Kapral, G.J., Grosse-Kunstleve, R.W., *et al.* (2010). PHENIX: a comprehensive Python-based system for macromolecular structure solution. Acta Crystallogr. D Biol. Crystallogr. 66, 213-221.

Emsley, P., and Cowtan, K. (2004). Coot: model-building tools for molecular graphics. Acta Crystallogr. D Biol. Crystallogr. 60, 2126-2132.

Otwinowski, Z., and Minor, W. (1997). Processing of X-ray diffraction data collected in oscillation mode. Methods Enzymol. 276, 307-326.

Supplemental Table SI Data collection and refinement statistics

Crystal name	**Wild-type-crystal**	**Se-Met-crystal**
Unit cell		
Space group	*H* 3 2	*H* 3 2
a, ***b***, ***c*** (Å)	***a*** = ***b*** = 114.311	***a*** = ***b*** = 114.351
	c = 183.479	***c*** = 183.199
Molecule/ asu	1	1
Wavelength (Å)	0.9791	0.9791
Resolution range (Å)	50- 2.2 (2.28- 2.2)	50-2.6 (2.69-2.6)
No. of unique reflections	23,421 (2,294)	14,409(1,408)
Redundancy	11.1(11.3)[a]	22.3(22.4)[a]
$\boldsymbol{R}_{\mathrm{sym}}$ (%)[b]	6.4(48.3) [a]	8.1 (48.7)[a]
$\boldsymbol{I/\sigma}$	23.52 (5.44) [a]	72.56(9.91)[a]
Completeness (%)	99.02 (98.96) [a]	99.9 (98.6) [a]
Refinement		
Resolution range (Å)	26.96-2.2	
$\boldsymbol{R}_{\mathrm{crystal}}$ (%)[c]	21.60(26.38)	
$\boldsymbol{R}_{\mathrm{free}}$ (%)[d]	24.42 (28.26)	
$\mathrm{RMSD_{bond}}$ (Å)	0.009	
$\mathrm{RMSD_{angle}}$(°)	1.26	
Number of		
non-hydrogen atoms	2,592	
macromolecules	2,517	
protein residues	324	
water	62	
Residues in (%)		
Ramachandran favored	97.0	
Ramachandran allowed	2.68	
Ramachandran outliers	0.32	
Average B factor ($Å^2$)	54.70	
macromolecules	54.60	
Solvent	56.70	

[a] the highest resolution shell;

[b] $R_{sym} = \sum_j \left| \langle I \rangle - I_j \right| \Big/ \sum \langle I \rangle$;

[c] $\boldsymbol{R}_{\mathrm{crystal}} = \sum_{hkl} \left| F_{obs} - F_{calc} \right| \Big/ \sum_{hkl} F_{obs}$;

[d] $\boldsymbol{R}_{\mathrm{free}}$, calculated the same as $\boldsymbol{R}_{\mathrm{crystal}}$, but from a test set containing 5% of data excluded from the refinement calculation.

Supplemental Figure 1

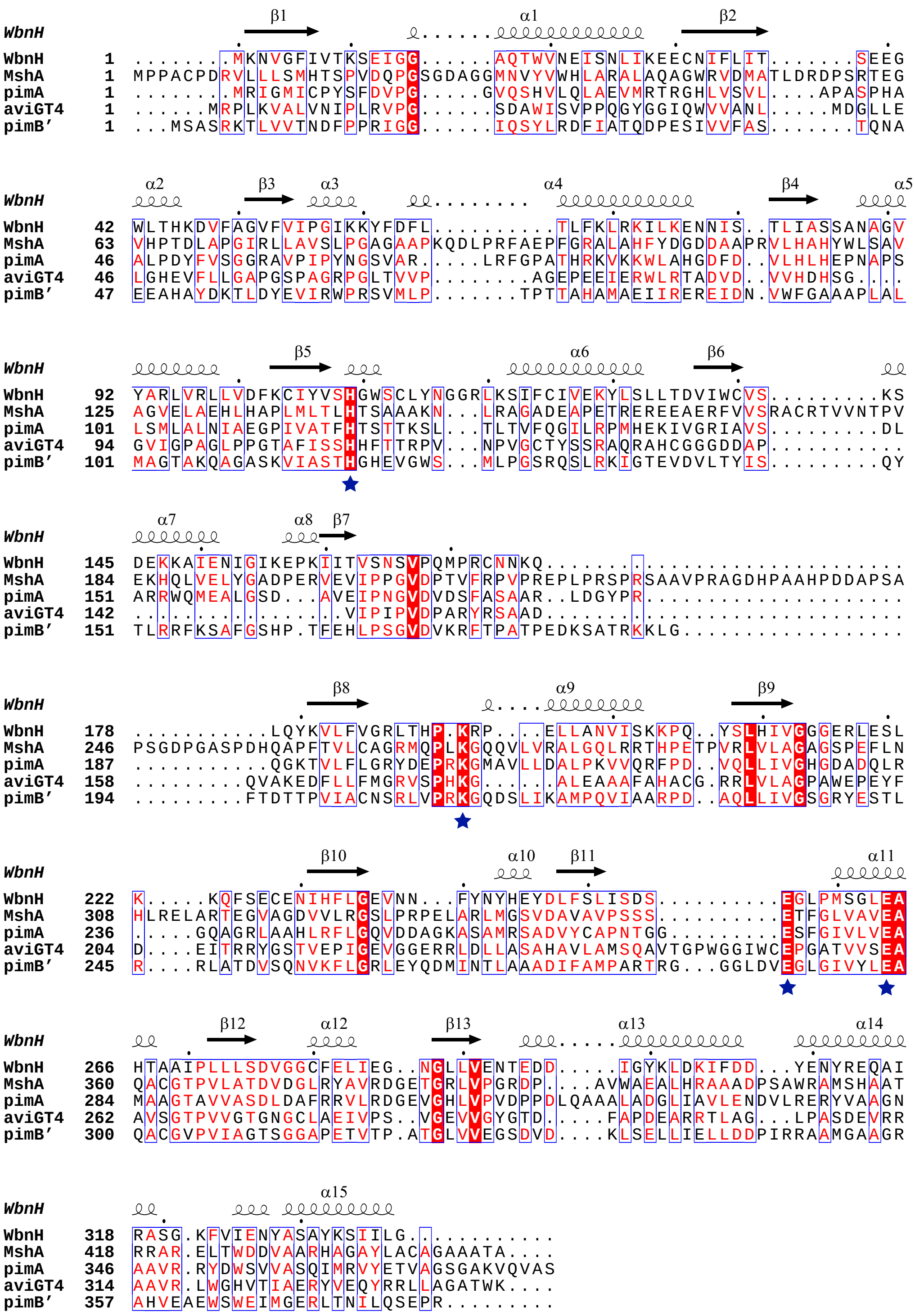

Sequence alignment of WbnH with other glycosyltransferases. The amino acid sequences of some GT-B family members (i.e., WbnH; the *Corynebacterium glutamicum* glycosyltransferase MshA; the phosphatidylinositol mannosyltransferase PimA from *Mycobacteria*; the glycosyltransferase aviGT4 from *Streptomyces viridochromogenes*, which is involved in avilamycin A biosynthesis; and the phosphatidylinositol mannosyltransferase PimB' from *Corynebacterium glutamicum*) were aligned together. The conserved residues are highlighted in red. The secondary structure is drawn based on the WbnH structure. The conserved residues for substrate binding are designated by blue stars.

Supplemental Figure 2

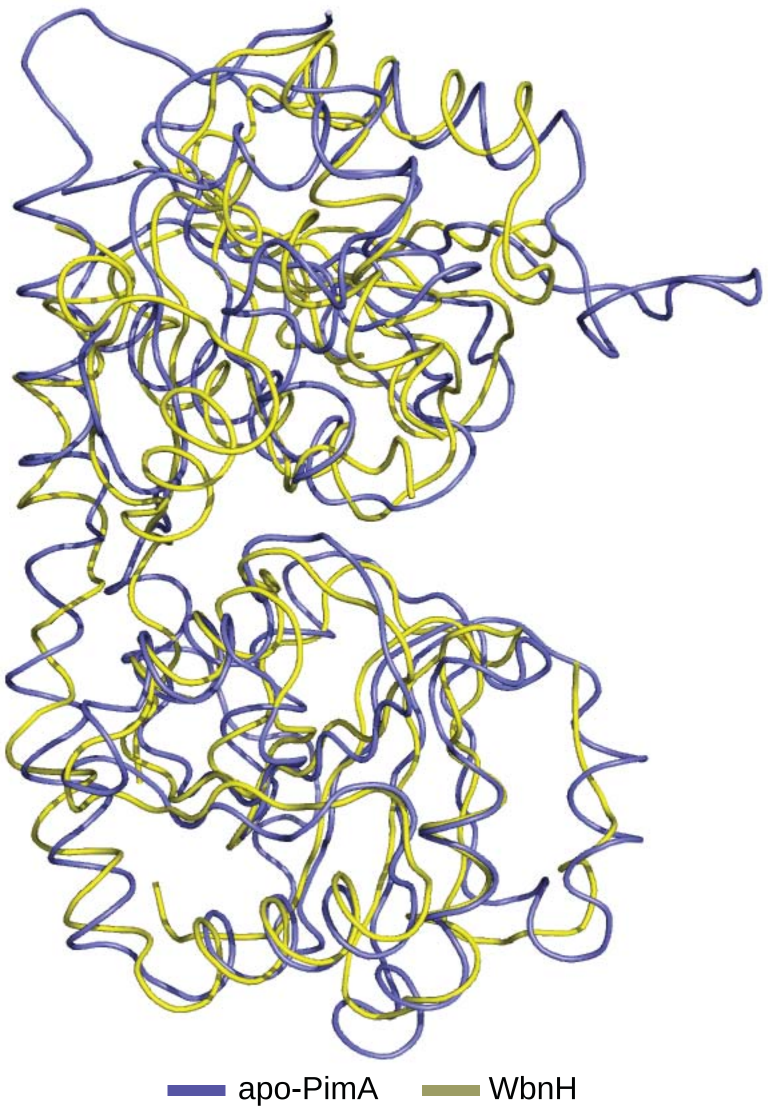

Structural comparison of WbnH and apo-PimA. The PDB code of apo-PimA is 4N9W. WbnH is presented in yellow, and apo-PimA is presented in blue.

Supplemental Figure 3

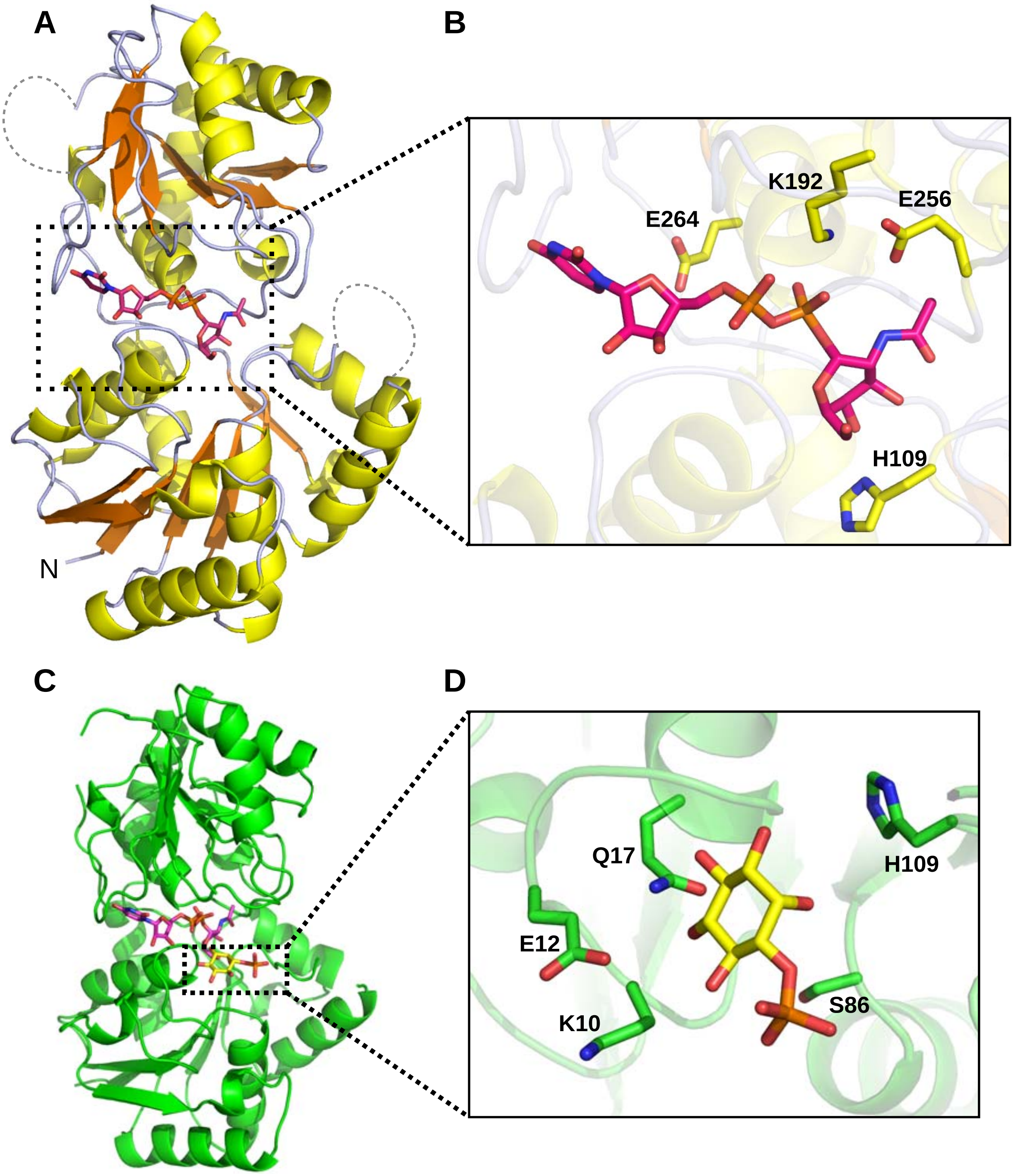

Model of WbnH with the donor and the acceptor. (A) Model of WbnH binding to the donor substrate UDP-GalNAc. (B) Magnified view of the active center. The side chains of four conserved residues (i.e., His109, Lys192, Glu256, and Glu264) that surround the proposed position of UDP-GalNAc are represented as yellow sticks. (C) Model of WbnH binding to the donor substrate UDP-GalNAc (magenta carbons) and the acceptor analog 1-L-Ins-1-P (yellow carbons). (D) Magnified view of the proposed acceptor binding site. The side chains of five residues that surround the acceptor analog are represented as green sticks.
